# Pre-Clinical Assessment of SAR442257, a CD38/CD3xCD28 Trispecific T Cell Engager in Treatment of Relapsed/Refractory Multiple Myeloma

**DOI:** 10.3390/cells13100879

**Published:** 2024-05-20

**Authors:** Anna Luise Grab, Peter S. Kim, Lukas John, Kamlesh Bisht, Hongfang Wang, Anja Baumann, Helgi Van de Velde, Irene Sarkar, Debarati Shome, Philipp Reichert, Calin Manta, Stefanie Gryzik, Rogier M. Reijmers, Niels Weinhold, Marc S. Raab

**Affiliations:** 1 Heidelberg Myeloma Center, Department of Medicine V, Medical Faculty Heidelberg and University Hospital, Heidelberg University, Im Neuenheimer Feld 410, 69120 Heidelberg, Germany; annaluise.grab@med.uni-heidelberg.de (A.L.G.); calin-petru.manta@med.uni-heidelberg.de (C.M.); stefanie.gryzik@med.uni-heidelberg.de (S.G.); niels.weinhold@med.uni-heidelberg.de (N.W.); 2 Clinical Cooperation Unit Molecular Hematology/Oncology, German Cancer Research Center (DKFZ), 69120 Heidelberg, Germany; 3 Sanofi Research and Development, Sanofi North America, Cambridge, MA 02141, USAkamlesh.bisht@sanofi.com (K.B.); hongfang.wang@sanofi.com (H.W.); helgi.vandevelde@sanofi.com (H.V.d.V.); 4 LUMICKS, 1059 CM Amsterdam, The Netherlands; i.sarkar@lumicks.com (I.S.); d.shome@lumicks.com (D.S.); r.reijmers@lumicks.com (R.M.R.); 5 GMMG Central Study Lab, Biobank, University Hospital Heidelberg, 69120 Heidelberg, Germany; philipp.reichert@med.uni-heidelberg.de

**Keywords:** T cell engager, cell avidity, refractory multiple myeloma, microenvironment

## Abstract

Current treatment strategies for multiple myeloma (MM) are highly effective, but most patients develop relapsed/refractory disease (RRMM). The anti-CD38/CD3xCD28 trispecific antibody SAR442257 targets CD38 and CD28 on MM cells and co-stimulates CD3 and CD28 on T cells (TCs). We evaluated different key aspects such as MM cells and T cells avidity interaction, tumor killing, and biomarkers for drug potency in three distinct cohorts of RRMM patients. We found that a significantly higher proportion of RRMM patients (86%) exhibited aberrant co-expression of CD28 compared to newly diagnosed MM (NDMM) patients (19%). Furthermore, SAR442257 mediated significantly higher TC activation, resulting in enhanced MM killing compared to bispecific functional knockout controls for all relapse cohorts (Pearson’s r = 0.7). Finally, patients refractory to anti-CD38 therapy had higher levels of TGF-β (up to 20-fold) compared to other cohorts. This can limit the activity of SAR442257. Vactoserib, a TGF-β inhibitor, was able to mitigate this effect and restore sensitivity to SAR442257 in these experiments. In conclusion, SAR442257 has high potential for enhancing TC cytotoxicity by co-targeting CD38 and CD28 on MM and CD3/CD28 on T cells.

## 1. Introduction

Multiple myeloma (MM) is a hematological cancer characterized by the accumulation of clonal plasma cells in the bone marrow. Despite therapeutic improvements to cure MM, most patients gain drug resistance and will require additional therapy [1,2,3,4,5,6,7]. Immunotherapy plays an important role in the treatment of MM, with agents such as elotuzumab (ELO), daratumumab, isatuximab, or bispecific antibodies for TC engagement being used to significantly improve survival rates. ELO is a monoclonal antibody that targets the SLAMF7 protein [8] and activates natural killer (NK) cells [9]. It is approved in combination with pomalidomide (POM) and dexamethasone after two prior lines of therapy [10,11]. Daratumumab and isatuximab are monoclonal antibodies directed against CD38 on MM cells and approved in various combinations [12]. Furthermore, bispecific antibodies for TC engagement [13] have recently shown convincing clinical efficacy profiles in RRMM [14,15], i.e., BCMA-CD3-antibodies [16,17,18] and GPRC5D-CD3 antibodies have shown high response rates with ORRs of 64–74% [19]. Resistance mechanisms against BCMA-targeted bispecific antibodies for TC engagement include high levels of soluble BCMA, reduced expression of surface BCMA [20,21], loss of target epitope expression, as well as exhaustion and dysfunction of TCs on and before therapy [7,22,23]. In order to overcome these issues, Wu et al. [24] described the development of SAR442257, a trispecific antibody that interacts with CD38, CD3, and CD28 to enhance T cell activation and tumor targeting. SAR442257 binding to CD3 induces transcriptional activation and downstream effector TC functions, enhanced by CD28 co-stimulation [24,25]. The anti-CD38 arm directs T cells to myeloma cells. Aberrant expression of CD28 has been observed on multiple myeloma cells, is associated with disease progression [4,26,27,28,29,30], and can be associated with a poor prognosis [31]. It may also confer a survival advantage, enabling MM cells to better withstand treatment and leading to their selective expansion [2]. Additionally, the aberrant expression of CD28 on myeloma cells is associated with the progression to stroma-independent disease [32]. Targeting CD28 may help to overcome these poor prognostic factors and can enhance MM cell recognition with such a trispecific antibody [24,33,34]. In vivo administration of this antibody suppressed myeloma growth in a humanized mouse model and stimulated memory/effector T cell proliferation, as well as reduced regulatory T cells in non-human primates at well-tolerated doses [24,35]. Therefore, targeting CD28 in addition to CD38 and CD3 aims to enhance the potency and durability of the T cell response [24]. The engagement of CD3 and CD28 provides efficient T cell activation and survival, aiming at overcoming T cell exhaustion that has been described to be associated with disease relapse with novel immunotherapies [24].

We hypothesized that SAR442257 might provide significant therapeutic advantages through the co-stimulatory signal mediated by CD28 and the dual targeting of CD38/CD28 on MM cells. To test this hypothesis, we investigated two key factors, i.e., (i) cell–cell avidity enhancement in the presence of the trispecific antibody SAR442257 compared to bispecific knockout controls (synthesized by Wu et al. [24] anti-CD38/CD3, anti-CD28/CD3, or monospecific anti-CD38 antibodies) and (ii) biomarkers for drug potency. Therefore, we focused on NDMM and three RRMM patient cohorts, i.e., refractory to prior therapy with anti-CD38 antibody-based combinations (daratumumab or isatuximab), bispecific antibodies for TC engagement, and immediate ELO/POM pretreatment. We assessed the CD28 target expression in these cohorts and the cytotoxic activity of TCs. Our findings reveal that the potency of the trispecific antibody in inducing TC cytotoxic activity is significantly higher compared to bi- and monospecific antibodies. We highlight that RRMM patients can be grouped into responders and non-responders impacted by T cell activation and TGF-β levels. Vactoserib, a TGF-β inhibitor [36], was evaluated as a potential combination therapy to counteract TGF-β-mediated TC exhaustion. The trispecific antibody SAR442257 shows promise for relapsed/refractory multiple myeloma by enhancing TC activation against tumor cells.

## 2. Materials and Methods

### 2.1. Cell Culture

Human multiple myeloma cell lines (HMCL), i.e., MM1S, U266, AMO, AMO-TP53, RPMI 8226, INA-6, OPM-2, LP-1, KMS-11BM, and KMS-11, were purchased from DSMZ (Braunschweig, Germany). Different HMCLs were used, showing varying surface expressions of CD28 and CD38.

Primary MM cells and bone marrow microenvironment cells: Bone marrow aspirates were collected from 100 NDMM and 40 RRMM patients 1 month after relapse to treatment of daratumumab, bispecific antibodies for TC engagement, or pomalidomide/elotuzumab after written informed consent as approved by the ethics committee of the University of Heidelberg (S-096/2017). Myeloma cells were purified from fresh bone marrow aspirates using density centrifugation followed by CD138-purification with anti-CD138 microbeads (Miltenyi Biotec, Bergisch Gladbach, Germany) and autoMACS Pro (Miltenyi Biotec, Bergisch Gladbach, Germany) as described previously [37]. For healthy donor samples, CD3-positive TCs were isolated from 9 peripheral blood buffy coats using CD3 MicroBeads (Milteney GmbH, Bergisch Gladbach, Germany) according to the manufacturer’s recommendation. Immediately after the purification steps, MM cells and bone marrow microenvironment cells were used for functional assays. TCs were pooled to avoid donor-specific responses. Cells were cultured in complete medium (RPMI 1640 GlutaMAX Media; Gibco, ThermoFisher, Darmstadt, Germany), supplemented with 10% heat-inactivated fetal bovine serum (FBS; Gibco, ThermoFisher, Darmstadt, Germany), at 37 °C in a humidified incubator under 5% CO_2_. 

SAR442257 and knockout controls (bispecific anti-CD38/CD3, anti-CD28/CD3, monospecific anti-CD38 antibodies, triple knockout as negative control) were obtained from Sanofi (Cambridge, MA, USA) as described previously [24].

### 2.2. Cell Binding Avidity Assays

MM cells adhered to microfluidic chips, and TCs were added together with the trispecific antibody or respective knockout controls to allow cell–cell binding. The cellular binding partners were separated by a linearly increasing force, and the frequency of bound TCs was detected as a function of the applied force.

This measurement was performed in 5 steps (Figure S1a) according to [38]. Avidity was measured with the z-Movi Cell Avidity Analyzer (Lumicks, Amsterdam, The Netherlands) using microfluidic chips that were coated with poly-L-lysine. After rehydration of the chips with warm RPMI 1640 media, cells were seeded at a density of 10^6^ cells/mL in serum-free medium. They were incubated for 30 min, followed by exchange with complete medium, and incubated for another 1.5 h to form a confluent monolayer. In the meantime, effector TCs were stained with the Celltrace Far Red Cell Proliferation Kit (ThermoFisher, Darmstadt, Germany, catalog C34564) according to the kit instructions. Each chip was mounted on the z-Movi Cell Avidity Analyzer (Lumicks, Amsterdam, The Netherlands). Stained TCs were mixed 1:1 with the relevant antibody and flown inside the chip at a density of 10^6^ cells/mL. They were incubated for 7.5 min with the monolayer in the presence of SAR442257 (1 nM, 10 nM, 100 nM, and 1000 nM) and corresponding bispecific controls, CD28/CD3-, CD38/CD3-, CD38-antibody, or triple-knockout antibody. Functionalized TCs recognize MM cells, followed by cell binding. After cell–cell contact was formed, the piezo element on top of the chamber generated an acoustic force. The acoustic force increased linearly over time, and with increasing force, more TCs are separated from their MM binding partners as the acoustic force exceeds their binding strength. The resulting avidity curves show the frequency of bound TCs as a function of applied force. Cell avidity experiments and analysis were conducted according to manufacturer recommendations. Cell detachment was analyzed using Ocean software version 2. 

### 2.3. Cytotoxicity Assays and TC Proliferation Assays

The in vitro testing of SAR442257 was conducted with TCs and CD38+ myeloma cell lines or with TC and autologous tumor cells isolated from the patient’s bone marrow (effector cells/tumor cells (E:T) ratio = 10:1). The killing assay consisted of co-cultures of CD138+ MM cells and their natural bone marrow microenvironment (CD138 negative fraction) incubated in the presence of an anti-CD38/CD3xCD28 trispecific antibody and its variants with a concentration range of 16 nM to 0.01 nM. Donor-derived TCs were added to the MM cells in a ratio of 10:1. For bone marrow MM cell frequencies below 0.9%, we used autologous TCs with E:T = 40 ± 13. Viability was assessed with PI staining after at least 24 h of co-culture, according to previously published protocols [37]. Measurements were performed using BD FACSAccuri.

In order to investigate the TGF-β effect on SAR442257 potency, we pre-exposed TCs from healthy donors to 10 pg/mL TGF-β for 24 h before adding them to MM1.S cells or primary MM cells from RRMM patients with a density of 10^6^ cells/mL. Moreover, 1 nM of SAR442257 was added for 24 h to the co-culture before harvesting the cells to allow flow cytometry (FACS) measurement of MM cell viability. For negative control, we used the TGF-β1 inhibitor vactoserib [36] (Selleckchem, Cologne, Germany). The cell culture medium of control cells was supplemented with vactoserib at a 1 nM concentration for 24 h. 

### 2.4. Cytokine Analysis

Cell-free supernatants from cocultures of primary MM cells and their natural bone marrow microenvironment at 24 h were analyzed for cytokine expression with the Human Essential Immune Response Panel (Biolegend GmbH, Amsterdam, The Netherlands) according to the manufacturer’s recommendation.

### 2.5. FACS Analysis 

We used FACS to quantify cell phenotyping and target expression in myeloma cell lines and patient samples. Cell-surface CD38 and CD28 density levels were measured on 40 RRMM and 100 NDMM patients by flow cytometry using a QIFIKIT (Agilent Dako, Glostrup, Denmark; catalog number K007811) according to customer protocol [39]. To normalize CD28 and CD38 target expression levels, we measured a calibration curve using QiFIKIT beads. The beads, which contain known amounts of fluorescent dye, were run on the same day as the cell samples, with the same PMT (photomultiplier tube) voltage and compensation settings, following the manufacturer’s staining protocol [39]. The mean fluorescence intensity of CD28 on MM cells was normalized to the CD28 expression in the patient’s bone marrow microenvironment (CD138 negative fraction). Normalized mean fluorescence values above 1 were interpreted as weakly positive and above 1.4 as highly positive [28]. All measurements were performed on a BD FACS Symphony A3 flow cytometer.

Immune subsets in patients’ bone marrow aspirates were analyzed using multicolor FACS phenotyping (Table 1), i.e., TCs, B cells, and NK cells, as well as TC activation/exhaustion markers in RRMM patients’ bone marrow. The staining reagents included the following antibodies: CD45-PacB, PD-1-PE-Cy7, CD3-APC-H7, CD4-BV510, CD8-PerCPCCy5.5, HLA-DR-FITC, CD57-APC, KI67-PE, CD25-PE-Cy7, CD69-APC, CD127-PE, CD45RA-BV421, CD27-PE-Cy7, Granzyme B-FITC, CCR7-APC, Perforin-PE, CD14-BV510, CD16-PerCP-Cy™5.5, CD56-APC, CD138-V500-C, CD20-PerCP-Cy™5.5, and CD19-PE-Cy™7, transcription factor buffer set, brilliant stain buffer plus (all reagents were obtained unless stated otherwise from BD, Heidelberg, Germany) and CD28-FITC (Clone 28.2 antibody; BioLegend, Amsterdam, The Netherlands). TC degranulation was accessed by CD107a-APC after adding GolgiStop (BD) to culture for 2 h according to the manufacturer’s protocol. Cell viability was determined using PI 50 µg/mL (BD, Heidelberg, Germany) with a dilution of 1:100 and an incubation time of 1 min.

### 2.6. Quantification and Statistical Analysis

All experiments were performed in at least two replicates and repeated for at least two patient-derived cells and MM cell lines. All results are represented as the mean ± SEM for the indicated number of observations. Data analysis and fitting were carried out using OriginPro2021 and Microsoft Excel. Significant differences between two groups were tested with two-tailed paired and unpaired Student’s *t*-tests with * *p* < 0.05, ** *p* < 0.01, *** *p* < 0.001. We evaluated the linear correlations between CD28 and CD38 target expression, as well as the linear correlations of drug response and TC activation and the correlations between target expression and MM cell death, using the Pearson correlation coefficient. The Pearson correlation measures the strength and direction of the linear relationship between two variables, allowing for comparison of the degree of linear association between drug response profiles across different cell lines and patients [40]. Statistical details have been provided in the figures and figure legends.

## 3. Results

We investigated the CD28 expression on 10 MM cell lines and plasma cells from 100 NDMM and 40 RRMM patients, as well as the potency of the trispecific antibody SAR442257 (anti-CD38/CD3xCD28) on primary samples from RRMM patients.

### 3.1. SAR442257 Enhances Cell Avidity and Tumor Killing in Human Myeloma Cell Lines

**Figure 1:**
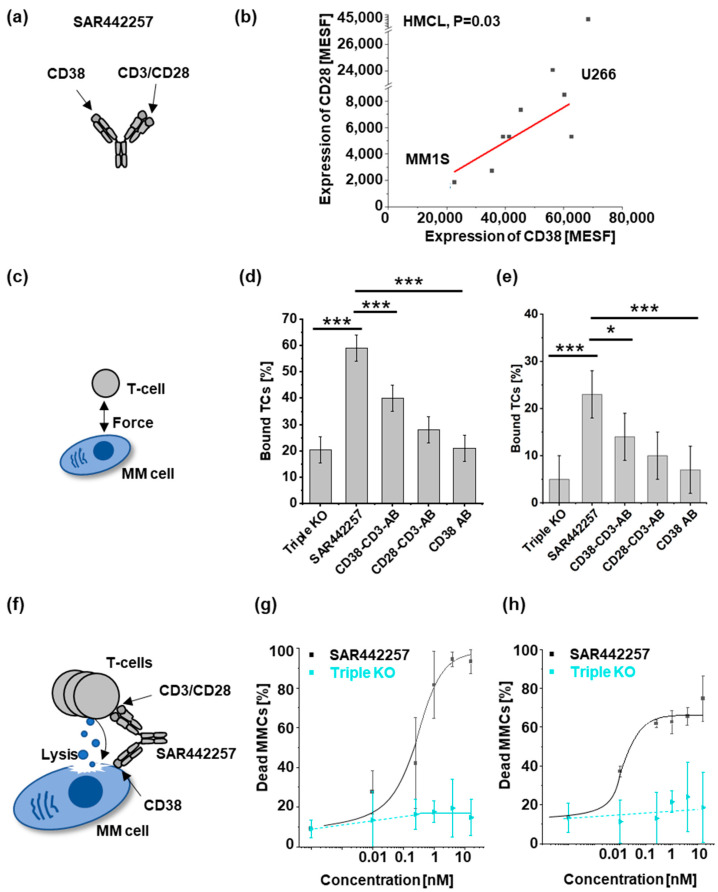
Characterization of SAR442257 potency in human multiple myeloma cell lines (HMCLs): (**a**) SAR442257 geometry; (**b**) correlation of target expression (in molecules of equivalent soluble fluorochrome (MESF)) of CD38 and CD28 on 10 HMCL. We measured CD38 and CD28 target expression and found a linear relationship with Pearson’s r = 0.7, *p* = 0.03. (**c**–**e**) Cellular avidity ranks as a percentage of TCs bound to MM cells by indicated TC engagers (triple knockout (KO), SAR442257, CD38-CD3-antibody (AB), CD28-CD3-AB, CD38 AB) for U266 (**d**) and MM1S (**e**). (**f**–**h**) Dose response curves of SAR442257 or triple KO control in (**g**) U266 and (**h**) MM1S with healthy donor TCs (E:T = 10:1). (Significance was determined using TTEST, * *p* < 0.05, *** *p* < 0.001).

The trispecific antibody SAR442257 binds to CD38, CD3, and CD28 to enable both tumor targeting and TC activation (Figure 1a). We assessed the CD38 and CD28 target expression levels of 10 HMCLs (Figure 1b), the enhancement of cell avidity (Figure 1c–e), and the killing efficiency (Figure 1f–h). 

Both target molecules, CD28 and CD38, were detectable on all HMCLs (Figure 1b). CD28 receptor densities (RD) were quantified by fluorescence intensity in molecules of soluble fluorochromes (MESF) and showed significant correlation to CD38 RD (Figure 1b, *p* = 0.03, Pearson’s r = 0.74). MM1S and U266 were chosen for further analyses as representatives of CD28/CD38 low- and high-expressing cell lines, respectively. 

Next, we investigated differences in cellular avidity mediated by trispecific antibodies and bispecific knockout controls. We observed that TC-to-MM cell binding increased as a function of drug concentration and was further investigated at its maximum (Figure S1b–e). The fraction of TCs bound to MM cells after incubation with the trispecific antibody was significantly higher than for knockout controls (bispecific, monospecific antibody, triple knockout as negative control) (Figure 1d,e). Specifically, the highest fraction of bound TCs to MM cell targets was observed for the trispecific antibody in both cell lines, with U266 showing a higher absolute binding fraction compared to MM1.S. For both cell lines, we observed that SAR442257 increased the frequency of bound TCs significantly by a factor of about 1.5 compared to the CD38-CD3 bispecific antibody (CD38-CD3-AB). 

Next, we asked whether reduced target expression decreases drug potency. Comparing MM1.S and U266 for the dose-dependent efficacy of SAR442257, we found EC50 and EC75 were 0.02 nM and 16 nM for MM1.S and 0.2 nM and 0.5 nM for U266, respectively (Figure 1f–h). Next, 10 HMCL were assessed for viability at a 1 nM concentration of SAR442257 (Figure S2a,b). SAR442257 significantly reduced the viability of 10/10 myeloma cell lines tested, ranging from 58% to 97% reduction within 24 h. Drug potency did not significantly correlate with target expression (Pearson’s r = 0.54, t-test *p* = 0.4 for CD28, and *p* = 0.1 for CD38). 

### 3.2. Activity of SAR442257 in RRMM Patient Cohorts

**Figure 2:**
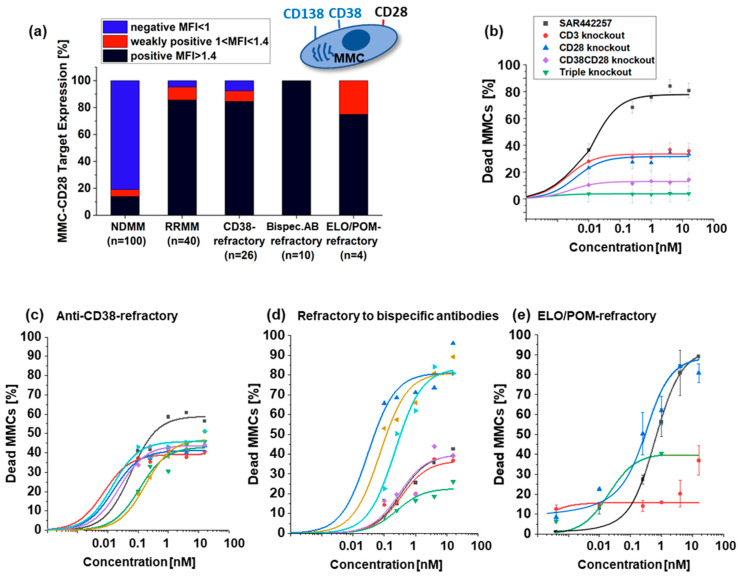
(**a**) Percentage of CD28 positive NDMM (n = 100) and the mean values of CD28 positive RRMM (n = 40), anti-CD38-refractory (n = 26), bispecific antibodies for TC engagement-refractory (n = 10), and ELO/POM-refractory (n = 4) patients (black bar indicates CD28 positive target expression, red bar weakly positive and blue bar CD28 negative target expression). (**b**) Mean value of dose-response curves of indicated TC engagers for 40 investigated samples from refractory MM patients. We show the percentage of dead MMCs after contact to SAR442257 (black), CD3 knockout antibody (red), CD28 knockout (blue) CD38CD28 knockout (violet) and triple knockout (green). (**c**–**e**) Dose response curves for patients with different pretreatment (**c**) anti-CD38 antibody refractory, (**d**) refractory to bispecific antibodies for TC engagement, and (**e**) ELO/POM refractory patients. Each patient is represented in a different color.

To assess the efficacy of SAR442257 in primary MM samples, we first characterized samples from NDMM (n = 100) and RRMM (n = 40) patients, including RRMM patients from three cohorts, as follows: cohort 1: refractory to anti-CD38 antibodies (n = 26); cohort 2: refractory to bispecific antibodies for TC engagement (n = 10); or cohort 3: ELO/POM (n = 4), for aberrant expression of CD28 on primary MM cells using mean fluorescence intensity (MFI) by flow cytometry (Figure 2a). 

While only a few samples scored positive in the NDMM samples (19%), the majority of MM cells from RRMM patients aberrantly expressed CD28 (86%), making the dual CD/38/CD28 targeting of SAR442257 attractive for this challenging patient population.

To assess the ability of SAR442257 to overcome drug resistance, primary MM cells from three RRMM cohorts were cultured together with autologous TCs and exposed to increasing concentrations of SAR442257. At concentrations above 1 nM, the trispecific antibody-mediated MM cell death in 40/40 patients (Figure 2b–e) ranged from 15% to 80%, and EC50 was reached for 7/26 patients in cohort 1, 5/10 in cohort 2, and 2/4 in cohort 3 at 1 nM SAR442257. We compared the efficacy of the trispecific antibody to the respective knockout variants in a pooled analysis of all tested samples from RRMM patients. As shown in Figure 2b, targeting CD3/CD28/CD38 simultaneously achieved synergistic efficacy compared to the bispecific variants CD3/CD38 and CD3/CD28, respectively. However, across the patient cohorts, the following two response groups emerged: good responders with more than 50% dead MM cells at 1 nM and poor responders with an EC50 not reached at 1 nM. To address whether these two response groups are characterized by differences in target expression, we first compared the target density of CD38 and CD28 between the patient cohorts and did not see any significant differences in CD28 or CD38 expression (Figure S3e,f, Pearson’s r = 0.4, *p* > 0.05). Furthermore, we analyzed CD38/CD28-target expression of bone marrow microenvironment B cells, TCs, and NK cells in the three different cohorts (Figure S3) and found significantly more Treg cells in patients refractory to anti-CD38 antibodies (cohort 1), and the numbers of early activated TCs as well as NK cells were significantly reduced (*p* < 0.05). We did not detect any differences in target expression between response groups (Figure S3), and we suspect effector cells have impaired cellular immune function. 

### 3.3. Tumor Cell Killing Correlates with Induced TC Activation 

**Figure 3:**
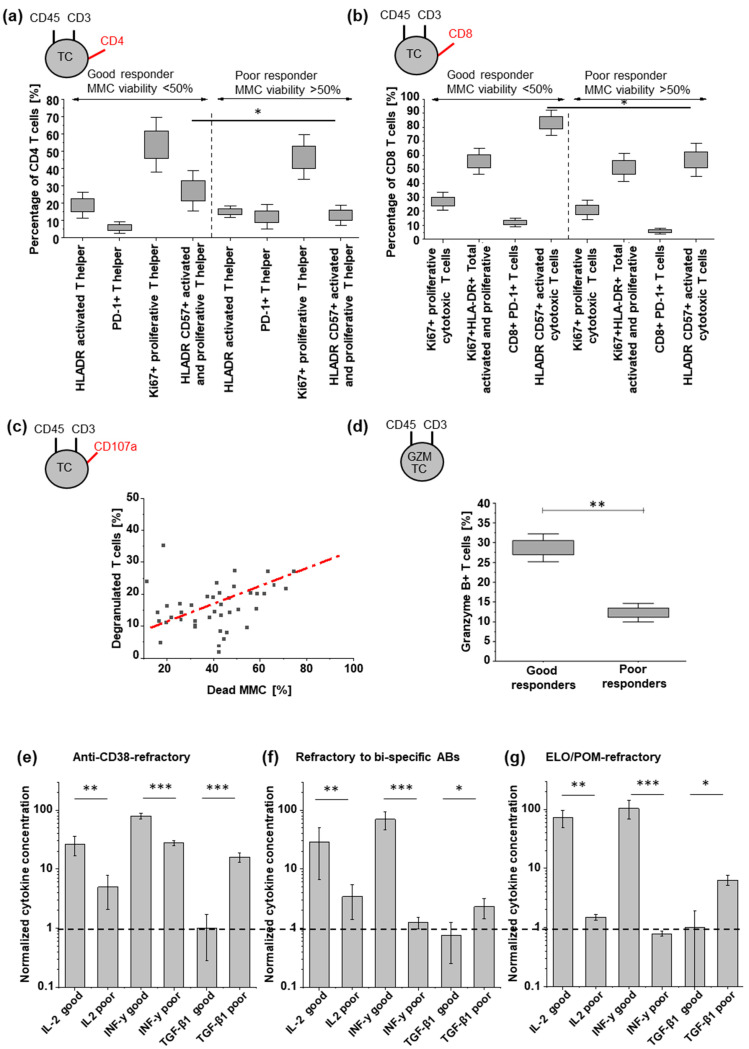
Characterization of CD4 and CD8 TC states, degranulation, and cytokine release. (**a**,**b**) TC subpopulations indicated by respective markers, according to good and poor response groups. (**c**) Tumor killing correlates with SAR442257-induced TC activation (Pearson’s r = 0.7). (**d**) frequency of granzyme B-positive TCs and (**e**–**g**) comparison of respective concentrations of IL2, INF-y, and TGF-β1 for good and poor responders after 24 h of exposure to SAR442257 (significance was determined using TTEST, * *p* < 0.05, ** *p* < 0.01, *** *p* < 0.001). The dotted line represents a normalized cytokine concentration of 1.

To assess differences in TC activation between responder groups, we analyzed CD4 and CD8-positive TCs for cellular proliferation, degranulation, and the release of cytokines after 24 h at 1 nM SAR442257 for good and poor responders. Significant differences between both groups were observed for CD4-positive activated and proliferative T helper cells and CD8-positive total activated cytotoxic TCs (Figure 3a,b). This effect is more pronounced in samples refractory to bispecific antibodies for TC engagement compared to anti-CD38-refractory RRMM patients (Figure S4). For all patients included in the study, we observed an induction of MM cell lysis by SAR442257 in correlation to TC degranulation (Pearson’s r = 0.7, Figure 3c). Next, we characterized Granzyme B-positive TCs. Good responders had 2.5-fold more Granzyme B-positive TC (Figure 3d). For cytokine release, we assessed the release of IL-2, INF-γ, and TGF-β1 (Figure 3e–g). IL2 and INF-y concentrations in supernatants after exposure to SAR442257 at 1 nM for 4 days were significantly higher in the good responders of all three patient cohorts. This indicates a higher TC activation in good responders, along with enhanced frequencies of cytotoxic and Granzyme B-positive TCs. In contrast, TGF-β levels were significantly higher for poor response in all patient cohorts and most pronounced in daratumumab-refractory patients.

### 3.4. TGF-β Can Reduce TC Engager Efficiency by Reducing T Cell Activation

**Figure 4:**
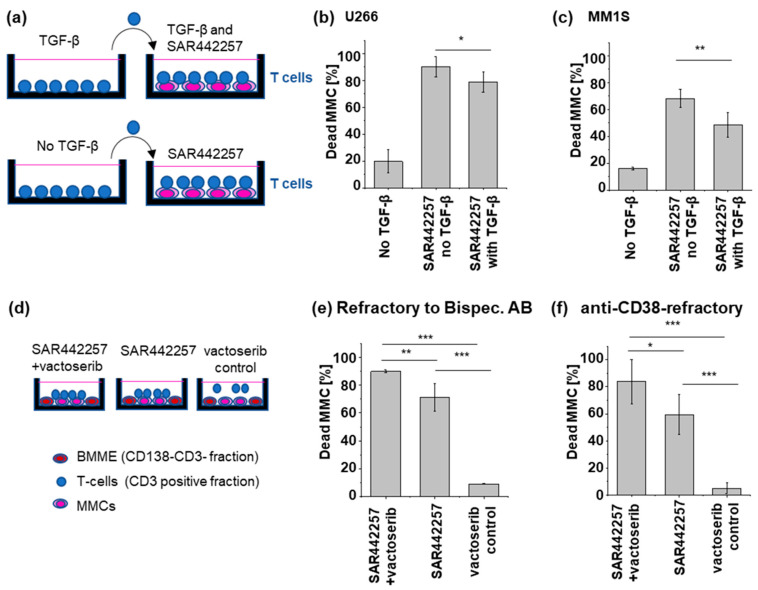
Presence of TGF-β in co-culture reduces TCE efficiency. (**a**) Sketch of the experimental setup (**b**,**c**) After a 24-hour culture in TGF-β-supplemented medium, healthy donor TCs are subsequently introduced into a co-culture with (**b**) U266 and (**c**) MM1S. TGF-β significantly reduced the drug potency of SAR442257. (**d**–**f**) Primary T cells were isolated from patient samples, and MM cells (MMCs) were co-cultured in the presence of the natural bone marrow microenvironment (BMME, CD138-CD3 fraction). Vactoserib, SAR442257, or a combination of vactoserib and SAR442257 were then added to the co-culture system. Vactoserib-SAR442257 combinations overcome TGF-β-induced T cell exhaustion. SAR442257 induced significantly higher MM cell killing in the presence of vactoserib in (**e**) bispecific antibodies for TC engagement and (**f**) anti-CD38-refractory samples. Viability was measured after 24 h of co-culture using FACS. All measurements were carried out in duplicates (significance was determined using TTEST, * *p* < 0.05, ** *p* < 0.01, *** *p* < 0.001).

By simultaneously engaging CD3 and CD28, the trispecific antibody is able to provide efficient TC stimulation. However, the TC stimulation can be reduced by inhibitory cytokines such as TGF-β, resulting in impaired MM cell killing. To further investigate the effect of TGF-β on TC activation in the presence of SAR442257, we pre-exposed TCs from healthy donors to 10 pg/mL TGF-β for 24 h before co-culturing them with U266 or MM1.S cells and 1 nM of SAR442257 for 24 h (Figure 4a). TGF-β reduced TC activation by SAR442257. Consequently, TGF-β leads to significantly lower trispecific antibody-mediated cell death in both cell lines (Figure 4b,c). To explore approaches to overcome TGF-β-mediated resistance, we used the TGF-β1 inhibitor vactoserib in combination with the trispecific antibody (Figure 4d–f). We observed a partial rescue of TGF-β-mediated reduction in the efficacy of SAR442257 by vactoserib in two primary MM cells refractory to bispecific antibodies for TC engagement (Figure 4e) and anti-CD38 therapy (Figure 4f).

## 4. Discussion

During the last decades, advances in the immunotherapy of RRMM have led to remarkable new therapies and improved progression-free survival [1,41]. One promising approach is the engagement of TCs by bispecific antibodies [42]. However, patients can develop primary and acquired resistance [16,43,44,45,46,47]. Wu et al. have developed SAR442257, a trispecific CD38/CD3xCD28 antibody, enabling TC co-stimulation by CD3 and CD28 as well as tumor targeting by CD28 and CD38. SAR442257 was designed by Wu et al. [24] to improve the activation and durability of the T cell response [24]. SAR442257 demonstrated antitumor activity in hematological malignancies [48], including MM, acute myeloid leukemia, chronic lymphocytic leukemia, and selected T and B cell lymphomas [24,48,49]. Here, we expanded the experimental cohort to RRMM primary samples. Furthermore, our study demonstrates the potential of SAR442257 in RRMM after immediate prior therapy with daratumumab, bispecific antibodies for TC engagement, and ELO/POM.

We investigated the role of target expression by elucidating differences between CD38 targeting and CD28 targeting. We quantified CD28 expression in myeloma cell lines and patient samples, including newly diagnosed and relapsed/refractory cases. We found that CD28 is expressed in a significant proportion of myeloma cells, particularly in relapsed/refractory disease. We found that the majority of RRMM patients expressed both targets, CD38 and CD28, on MM cells. We ranked CD28-null and CD38-null trispecific Abs for cell–cell binding force and killing efficiency. While CD38-CD3 bispecific Ab had a slightly reduced cell avidity in HMCL compared to CD28-CD3 bispecific Ab and lower killing efficiency on average in RRMM, we observed increased killing efficiency of CD28-CD3 bispecific Ab over CD38-CD3 bispecific Ab in the daratumumab relapse cohort. We suspect the CD28 binding of the antibody plays a critical role in TC co-stimulation and enhanced MM cell recognition.

Wu et al. [24] have demonstrated that CD28 expression in multiple myeloma cells increases susceptibility to cytolysis. This is of major importance, especially since the frequency of CD28 expression is increased during myeloma progression [4,30,31,32]. Others have investigated anti-CD28-monospecific antibodies for MM cell targeting and showed that anti-CD28 alone already induced significant suppression of MM cell proliferation [4]. This effect could actually be increased by trispecific antibodies [24,49] and manifests that CD28 on MM cells and TC co-stimulation can play an important role in overcoming drug resistance in RRMM, e.g., after daratumumab treatment, by improving tumor recognition and long-term TC activation [50,51]. Furthermore, we found that SAR442257 killing efficiency in HMCL increased with CD28 and CD38 target expression. This finding is similar to observations for mechanisms of resistance of TC-engaged drugs, which may involve downregulation of tumor-associated antigens resulting in tumor escape [24,52]. 

We also investigated the role of the immune subset composition in drug responses. Interestingly, all RRMM patients showed a significant response to SAR442257 and could be classified into two cohorts, i.e., good responders with complete MM cell lysis and poor responders, which showed a response up to EC50 at 1 nM of SAR442257. A good response was indicated by TCs with a high frequency of proliferation and activation states. More specifically, characteristics for good responders to SAR442257 with complete MM cell lysis were the pre-existence of activated and cytotoxic TC subsets in immune phenotyping as well as increased TC degranulation and IL-2 and Granzyme-B production. This finding shows the importance of CD28 targeting in agreement with the observations of Wu et al. [24] and is consistent with tumor immune escape to TC engager treatments, caused by not only the loss of target antigen but also the proportion of pre-existing exhausted-like CD8+ TCs [7]. 

We observed an increase in Tregs in daratumumab-refractory patients; however, the role of Tregs in MM has remained controversial [53]. Interestingly, it has been observed that marrow-infiltrating Tregs correlate with the presence of dysfunctional CD4+ PD-1+ cells [54]. Low effector CD4 (CD4eff)/Tregs ratio and increased frequency of PD-1-expressing CD4eff cells were independent predictors of early relapse to daratumumab treatment over and above conventional risk factors, such as genetic risk and depth of response [53]. Nevertheless, a subpopulation of CD38+ Treg was found to be more immunosuppressive than CD38− Treg and decreased in patients treated with daratumumab, suggesting an additional mechanism of action for this anti-CD38 antibody used to treat MM patients [53].

Besides cellular factors, soluble non-cellular components such as TGF-β can influence drug efficiency [55,56], which can be critical for intrinsic drug resistance by repressing anti-myeloma immunity and disease progression. Here, we observed increased TGF-β concentrations in patients with poor responses to SAR442257 because TGF-β significantly reduced TC activation and MM cell lysis. We investigated the TGF-β inhibitor vactoserib [57] to improve SAR442257 potency in those samples. 

We suspect it is necessary to stimulate TCs, especially for patients refractory to mono- and bispecific antibodies. In the combinatorial regime of SAR442257 and vactoserib, potent MM cell lysis was also seen in samples from heavily pretreated patients with daratumumab resistance. Other studies have focused on the expansion of TCs by CD47 targeting [58], CD55/CD59 inhibitors [59], or the enhancement of NK effector functions [12,60,61]. 

One limitation of the study is that it does not include a comprehensive evaluation of the safety profile [24,62] of the compound. In particular, the novel mechanism of action of SAR442257 cannot be investigated in traditional multiple myeloma models [24]. In addition to the activity on myeloma plasma cells, T cells may be engaged through all three targets, CD3, CD28, and CD38 [12,24,63,64,65,66]. While animal toxicology studies in non-human primates were reassuring [24], a first-in-human dose-escalation clinical trial is expected to provide the first information on the safety of this agent in humans.

## 5. Conclusions

SAR442257 demonstrated significantly higher killing of MM cells compared to bispecific functional knockout controls for all relapse cohorts, which correlated with enhanced TC activation and degranulation. This enhanced activity is attributed to the increased binding capacity of SAR442257 for RRMM due to its dual targeting of CD38 and CD28, compared to monospecific or bispecific CD38/CD3 antibodies.

Importantly, our study quantified the expression of CD28 on myeloma cell lines and patient samples, including newly diagnosed and relapsed/refractory cases. We found that CD28 is expressed in a significant proportion of myeloma cells, particularly in relapsed/refractory disease. This suggests that CD28 may be a promising target for the treatment of advanced myeloma.

In poor responders, additional agents, such as TGF-β inhibitors, could be combined with SAR442257 to further enhance its anti-myeloma activity. Overall, our findings support the idea that further investigation of SAR442257 as a potential immunotherapeutic approach for treating RRMM may be warranted.

## Figures and Tables

**Table 1:** Materials used in this study.

Material	Company	Cat. No.
SAR442257	Sanofi (MA, USA)	Not applicable (N/A)
CD45	Invitrogen (Basel, Switzerland)	MHCD4528
PD-1 (=CD279)	BD (Heidelberg, Germany)	560,652
CD3	BD (Heidelberg, Germany)	641,397
CD4	BD (Heidelberg, Germany)	562,970
CD8	BD (Heidelberg, Germany)	341,050
HLA-DR	BD (Heidelberg, Germany)	556,643
CD57	BD (Heidelberg, Germany)	560,845
KI67	BD (Heidelberg, Germany)	567,120
TIGIT	BioLegend (Amsterdam, The Netherlands)	372,709
CD25	BD (Heidelberg, Germany)	557,741
FOXP3	Bioscience/Affimetrix (Heidelberg, Germany)	11-4776-42
CD69	BD (Heidelberg, Germany)	560,711
CD127	ThermoFisher (Darmstadt, Germany)	BDB-557938
CD45RA	BD (Heidelberg, Germany)	740,083
CD27	BD (Heidelberg, Germany)	567,289
Granzyme	BD (Heidelberg, Germany)	558,905
CCR7	BD (Heidelberg, Germany)	566,762
Perforin	BD (Heidelberg, Germany)	556,437
4-1BB Cd137	BD (Heidelberg, Germany)	741,000
CD14	BD (Heidelberg, Germany)	740,163
CD16	BD (Heidelberg, Germany)	338,426
CD56	BD (Heidelberg, Germany)	555,518
CD28	BD (Heidelberg, Germany)	556,621
CD138	BD (Heidelberg, Germany)	650,660
CD20	BD (Heidelberg, Germany)	560,736
CD319	Biomol (Hamburg, Germany)	ABD-131901C1
CD28	BD (Heidelberg, Germany)	556,621
CD19	BD (Heidelberg, Germany)	557,835
CD107a	BD (Heidelberg, Germany)	555,800
anti-CD138	Miltenyi Biotec (Bergisch Gladbach, Germany)	130-051-301
Buffy coat	DRK Baden–Württemberg–Hessen GmbH (Mannheim, Germany)	N/A
Patient material	University Hospital Heidelberg (Heidelberg, Germany)	N/A
QiFIKIT beads	Dako (Glostrup, Denmark)	K007811
PE/R-Phycoerythrin Conjugation Kit	Abcam (Amsterdam, The Netherlands)	ab102918
Transcription Factor Buffer Set	BD (Heidelberg, Germany)	562,574
Brilliant Stain Buffer Plus	BD (Heidelberg, Germany)	566,385
Legendplex	BioLegend (Amsterdam, The Netherlands)	741,157
autoMACS Pro purification	Miltenyi Biotec (Bergisch Gladbach, Germany)	N/A
MM1S	DSMZ (Braunschweig, Germany)	N/A
U266	DSMZ (Braunschweig, Germany)	N/A
AMO	DSMZ (Braunschweig, Germany)	N/A
AMO-TP53	DSMZ (Braunschweig, Germany)	N/A
RPMI 8226	DSMZ (Braunschweig, Germany)	N/A
INA-6	DSMZ (Braunschweig, Germany)	N/A
OPM-2	DSMZ (Braunschweig, Germany)	N/A
LP-1	DSMZ (Braunschweig, Germany)	N/A
KMS-11BM	DSMZ (Braunschweig, Germany)	N/A
KMS-11	DSMZ (Braunschweig, Germany)	N/A
Origin	ADDITIVE GmbH (Friedrichsdorf, Germany)	N/A
Ocean software	LUMICKS (Amsterdam, The Netherlands)	N/A
RPMI 1640	Gibco (ThermoFisher, Darmstadt, Germany)	11875-093
FBS	Gibco (ThermoFisher, Darmstadt, Germany)	10270-106
Vactoserib	Selleckchem (Cologne, Germany)	S7530
TGF-β	Selleckchem (Cologne, Germany)	A2113

## Data Availability

Data are available on request from annaluise.grab@med.uni-heidelberg.de.

## Supplementary Materials

The following supporting information can be downloaded at: https://www.mdpi.com/article/10.3390/cells13100879/s1, Figure S1. Workflow and Titration for Cellular avidity experiments; Figure S2. Analysis of SAR442257 potency to induce HMCL death; Figure S3. CD38/CD28-target expression of bone marrow microenvironment; Figure S4. Characterization of CD4 and CD8 TC states, degranulation, and cytokine release.

## References

[1] Raje N., Mateos M.-V., Iida S., Reece D. (2023). Clinical evidence for immune-based strategies in early-line multiple myeloma: Current challenges in decision-making for subsequent therapy. Blood Cancer J..

[2] Mateo G., Castellanos M., Rasillo A., Gutierrez N.C., Montalban M.A., Martin M.L., Hernandez J.M., Lopez-Berges M.C., Montejano L., Blade J. (2005). Genetic abnormalities and patterns of antigenic expression in multiple myeloma. Clin. Cancer Res..

[3] Almeida J., Orfao A., Ocqueteau M., Mateo G., Corral M., Caballero M.D., Blade J., Moro M.J., Hernandez J., San Miguel J.F. (1999). High-sensitive immunophenotyping and DNA ploidy studies for the investigation of minimal residual disease in multiple myeloma. Br. J. Haematol..

[4] Bahlis N.J., King A.M., Kolonias D., Carlson L.M., Liu H.Y., Hussein M.A., Terebelo H.R., Byrne G.E., Levine B.L., Boise L.H. (2007). CD28-mediated regulation of multiple myeloma cell proliferation and survival. Blood.

[5] Chung D.J., Pronschinske K.B., Shyer J.A., Sharma S., Leung S., Curran S.A., Lesokhin A.M., Devlin S.M., Giralt S.A., Young J.W. (2016). T-cell Exhaustion in Multiple Myeloma Relapse after Autotransplant: Optimal Timing of Immunotherapy. Cancer Immunol. Res..

[6] Pilcher W., Thomas B.E., Bhasin S.S., Jayasinghe R.G., Rahman A.H., Kim-Schulze S., Gonzalez-Kozlova E., Kourelis T., Dhodapkar M.V., Vij R. (2021). Characterization of T-Cell Exhaustion in Rapid Progressing Multiple Myeloma Using Cross Center Scrna-Seq Study. Blood.

[7] Friedrich M.J., Neri P., Kehl N., Michel J., Steiger S., Kilian M., Leblay N., Maity R., Sankowski R., Lee H. (2023). The pre-existing T cell landscape determines the response to bispecific T cell engagers in multiple myeloma patients. Cancer Cell.

[8] Awwad M.H.S., Mahmoud A., Bruns H., Echchannaoui H., Kriegsmann K., Lutz R., Raab M.S., Bertsch U., Munder M., Jauch A. (2021). Selective elimination of immunosuppressive T cells in patients with multiple myeloma. Leukemia.

[9] Dimopoulos M.A., Richardson P., Lonial S. (2022). Treatment Options for Patients With Heavily Pretreated Relapsed and Refractory Multiple Myeloma. Clin. Lymphoma Myeloma Leuk..

[10] Ailawadhi S., Parrondo R.D., Laplant B., Alegria V.R., Elliott J.B., Sher T., Paulus A., Chapin D., Heslop K., Chanan-Khan A. (2022). Phase II Trial of Elotuzumab with Pomalidomide and Dexamethasone for Relapsed/Refractory Multiple Myeloma (RRMM) in the Post-Daratumumab Progression Setting. Blood.

[11] Bryant A., Ling S.C.W., Trieu S. (2021). Elotuzumab. Resistance to Targeted Therapies in Multiple Myeloma.

[12] Saltarella I., Desantis V., Melaccio A., Solimando A.G., Lamanuzzi A., Ria R., Storlazzi C.T., Mariggiò M.A., Vacca A., Frassanito M.A. (2020). Mechanisms of Resistance to Anti-CD38 Daratumumab in Multiple Myeloma. Cells.

[13] Caraccio C., Krishna S., Phillips D.J., Schürch C.M. (2020). Bispecific Antibodies for Multiple Myeloma: A Review of Targets, Drugs, Clinical Trials, and Future Directions. Front. Immunol..

[14] Usmani S.Z., Garfall A.L., van de Donk N., Nahi H., San-Miguel J.F., Oriol A., Rosinol L., Chari A., Bhutani M., Karlin L. (2021). Teclistamab, a B-cell maturation antigen x CD3 bispecific antibody, in patients with relapsed or refractory multiple myeloma (MajesTEC-1): A multicentre, open-label, single-arm, phase 1 study. Lancet.

[15] Sanchez L., Dardac A., Madduri D., Richard S., Richter J. (2021). B-cell maturation antigen (BCMA) in multiple myeloma: The new frontier of targeted therapies. Ther. Adv. Hematol..

[16] Seckinger A., Delgado J.A., Moser S., Moreno L., Neuber B., Grab A., Li J., Stagg N.J., Johnston J., Harris M.J. (2017). T-Cell Bispecific Antibodies Suppress Multiple Myeloma. Cancer Discov..

[17] Raab M.S., Cohen Y.C., Schjesvold F., Aardalen K., Oka A., Spencer A., Wermke M., Souza A.D., Kaufman J.L., Cafro A.M. (2023). Preclinical discovery and initial clinical data of WVT078, a BCMA × CD3 bispecific antibody. Leukemia.

[18] Kararoudi M.N., Nagai Y., Elmas E., Pereira M.D.F., Ali S.A., Imus P.H., Wethington D., Borrello I.M., Lee D.A., Ghiaur G. (2020). CD38 deletion of human primary NK cells eliminates daratumumab-induced fratricide and boosts their effector activity. Blood.

[19] Chari A., Minnema M.C., Berdeja J.G., Oriol A., van de Donk N., Rodríguez-Otero P., Askari E., Mateos M.V., Costa L.J., Caers J. (2022). Talquetamab, a T-Cell-Redirecting GPRC5D Bispecific Antibody for Multiple Myeloma. N. Engl. J. Med..

[20] Lee H., Durante M., Ahn S., Leblay N., Poorebrahim M., Maity R., Tilmont R., Barakat E., Jung D., Ziccheddu B. (2023). The Impact of Soluble BCMA and BCMA Gain on Anti-BCMA Immunotherapies in Multiple Myeloma. Blood.

[21] Laurent S.A., Hoffmann F.S., Kuhn P.H., Cheng Q., Chu Y., Schmidt-Supprian M., Hauck S.M., Schuh E., Krumbholz M., Rubsamen H. (2015). gamma-Secretase directly sheds the survival receptor BCMA from plasma cells. Nat. Commun..

[22] Sade-Feldman M., Yizhak K., Bjorgaard S.L., Ray J.P., de Boer C.G., Jenkins R.W., Lieb D.J., Chen J.H., Frederick D.T., Barzily-Rokni M. (2019). Defining T Cell States Associated with Response to Checkpoint Immunotherapy in Melanoma. Cell.

[23] Li H., van der Leun A.M., Yofe I., Lubling Y., Gelbard-Solodkin D., van Akkooi A.C.J., van den Braber M., Rozeman E.A., Haanen J., Blank C.U. (2019). Dysfunctional CD8 T Cells Form a Proliferative, Dynamically Regulated Compartment within Human Melanoma. Cell.

[24] Wu L., Seung E., Xu L., Rao E., Lord D.M., Wei R.R., Cortez-Retamozo V., Ospina B., Posternak V., Ulinski G. (2020). Trispecific antibodies enhance the therapeutic efficacy of tumor-directed T cells through T cell receptor co-stimulation. Nat. Cancer.

[25] Beyersdorf N., Kerkau T., Hünig T. (2015). CD28 co-stimulation in T-cell homeostasis: A recent perspective. Immunotargets Ther..

[26] Lee H., Ahn S., Maity R., Leblay N., Ziccheddu B., Truger M., Chojnacka M., Cirrincione A., Durante M., Tilmont R. (2023). Mechanisms of antigen escape from BCMA- or GPRC5D-targeted immunotherapies in multiple myeloma. Nat. Med..

[27] Samur M.K., Fulciniti M., Aktas Samur A., Bazarbachi A.H., Tai Y.-T., Prabhala R., Alonso A., Sperling A.S., Campbell T., Petrocca F. (2021). Biallelic loss of BCMA as a resistance mechanism to CAR T cell therapy in a patient with multiple myeloma. Nat. Commun..

[28] Pellat-Deceunynck C., Bataille R., Robillard N., Harousseau J.L., Rapp M.J., Juge-Morineau N., Wijdenes J., Amiot M. (1994). Expression of CD28 and CD40 in human myeloma cells: A comparative study with normal plasma cells. Blood.

[29] Robillard N., Jego G., Pellat-Deceunynck C., Pineau D., Puthier D., Mellerin M.P., Barille S., Rapp M.J., Harousseau J.L., Amiot M. (1998). CD28, a marker associated with tumoral expansion in multiple myeloma. Clin. Cancer Res..

[30] Shapiro V.S., Mollenauer M.N., Weiss A. (2001). Endogenous CD28 expressed on myeloma cells up-regulates interleukin-8 production: Implications for multiple myeloma progression. Blood.

[31] Utley A., Peng P., Liu W.S., Ventrone D., Lee K.P. (2018). CD28 Induces Metabolic Fitness in Multiple Myeloma for ROS-Dependent Survival. Blood.

[32] Nair J.R., Carlson L.M., Koorella C., Rozanski C.H., Byrne G.E., Bergsagel P.L., Shaughnessy J.P., Boise L.H., Chanan-Khan A., Lee K.P. (2011). CD28 expressed on malignant plasma cells induces a prosurvival and immunosuppressive microenvironment. J. Immunol..

[33] Garfall A.L., June C.H. (2019). Trispecific antibodies offer a third way forward for anticancer immunotherapy. Nature.

[34] Gantke T., Weichel M., Herbrecht C., Reusch U., Ellwanger K., Fucek I., Eser M., Muller T., Griep R., Molkenthin V. (2017). Trispecific antibodies for CD16A-directed NK cell engagement and dual-targeting of tumor cells. Protein Eng. Des. Sel..

[35] Abrams R.E., Pierre K., El-Murr N., Seung E., Wu L., Luna E., Mehta R., Li J., Larabi K., Ahmed M. (2022). Quantitative systems pharmacology modeling sheds light into the dose response relationship of a trispecific T cell engager in multiple myeloma. Sci. Rep..

[36] Malek E., Rana P.S., Swamydas M., Daunov M., Miyagi M., Murphy E., Ignatz-Hoover J.J., Metheny L., Seong Jin K., Driscoll J.J. (2023). Vactosertib, a novel TGF-β1 type I receptor kinase inhibitor, improves T-cell fitness: A single-arm, phase 1b trial in relapsed/refractory multiple myeloma. Blood.

[37] Grab A.L., Seckinger A., Horn P., Hose D., Cavalcanti-Adam E.A. (2019). Hyaluronan hydrogels delivering BMP-6 for local targeting of malignant plasma cells and osteogenic differentiation of mesenchymal stromal cells. Acta Biomater..

[38] Larson R.C., Kann M.C., Bailey S.R., Haradhvala N.J., Llopis P.M., Bouffard A.A., Scarfó I., Leick M.B., Grauwet K., Berger T.R. (2022). CAR T cell killing requires the IFNγR pathway in solid but not liquid tumours. Nature.

[39] Urlaub D., Watzl C. (2020). Coated Latex Beads as Artificial Cells for Quantitative Investigations of Receptor/Ligand Interactions. Curr. Protoc. Immunol..

[40] Herbst S.A., Kim V., Roider T., Schitter E.C., Bruch P.M., Liebers N., Kolb C., Knoll M., Lu J., Dreger P. (2023). Comparing the value of mono- vs coculture for high-throughput compound screening in hematological malignancies. Blood Adv..

[41] Bazarbachi A.H., Al Hamed R., Malard F., Harousseau J.-L., Mohty M. (2019). Relapsed refractory multiple myeloma: A comprehensive overview. Leukemia.

[42] Moreau P., Touzeau C. (2022). T-cell-redirecting bispecific antibodies in multiple myeloma: A revolution?. Blood.

[43] Esensten J.H., Helou Y.A., Chopra G., Weiss A., Bluestone J.A. (2016). CD28 Costimulation: From Mechanism to Therapy. Immunity.

[44] Hashim L., Faisal M.S., Iqbal M.A., Saeed H., Samhouri Y., Shahzad M., Khattak Z.E., Anwer F. (2021). Bispecific T-Cell Engager Antibodies in Multiple Myeloma- a Systematic Review of Phase 1 Clinical Trials. Blood.

[45] Buie L.W., Pecoraro J.J., Horvat T.Z., Daley R.J. (2015). Blinatumomab: A First-in-Class Bispecific T-Cell Engager for Precursor B-Cell Acute Lymphoblastic Leukemia. Ann. Pharmacother..

[46] Seckinger A., Delgado J.A., Moser S., Moreno L., Neuber B., Grab A., Lipp S., Merino J., Prosper F., Emde M. (2017). Target Expression, Generation, Preclinical Activity, and Pharmacokinetics of the BCMA-T Cell Bispecific Antibody EM801 for Multiple Myeloma Treatment. Cancer Cell.

[47] Alhallak K., Sun J., Jeske A., Park C., Yavner J., Bash H., Lubben B., Adebayo O., Khaskiah A., Azab A.K. (2021). Bispecific T Cell Engagers for the Treatment of Multiple Myeloma: Achievements and Challenges. Cancers.

[48] Dupuy A., Pelletier L., Giustiniani J., Kim P., Bisht K., Wang H., Van de Velde H.J., Haioun C., Gaulard P., Ortonne N. (2023). The CD38/CD3xCD28 Trispecific Antibody (SAR442257) Potentially Represents a Novel Therapeutic Strategy for Peripheral T-Cell Lymphomas. Blood.

[49] Green M.R., Reville P.K., Dai E., Sheikh I., Deng Q., Henderson J., Le C., Rojas E., Okwuchi C., Wilson A. (2023). SAR442257, a CD38/CD28/CD3 trispecific antibody, potentiates CAR T-cell activity against large B-cell lymphoma. Hematol. Oncol..

[50] Viola D., Dona A., Caserta E., Troadec E., Besi F., McDonald T., Ghoda L., Gunes E.G., Sanchez J.F., Khalife J. (2021). Daratumumab induces mechanisms of immune activation through CD38+ NK cell targeting. Leukemia.

[51] Richardson P.G., Facon T., Bensinger W.I., Leleu X., Campana F., Macé S., Chiron M., van de Velde H., Mikhael J. (2021). Predictive biomarkers with isatuximab plus pomalidomide and dexamethasone in relapsed/refractory multiple myeloma. Blood Cancer J..

[52] Chen L., Qian W., Pan F., Li D., Yu W., Tong L., Yang Y., Xu Q., Ding J., Dai R. (2024). A trispecific antibody induces potent tumor-directed T-cell activation and antitumor activity by CD3/CD28 co-engagement. Immunotherapy.

[53] Krejcik J., Casneuf T., Nijhof I.S., Verbist B., Bald J., Plesner T., Syed K., Liu K., van de Donk N.W., Weiss B.M. (2016). Daratumumab depletes CD38^+^ immune regulatory cells, promotes T-cell expansion, and skews T-cell repertoire in multiple myeloma. Blood.

[54] Alrasheed N., Lee L., Ghorani E., Henry J.Y., Conde L., Chin M., Galas-Filipowicz D., Furness A.J.S., Chavda S.J., Richards H. (2020). Marrow-Infiltrating Regulatory T Cells Correlate with the Presence of Dysfunctional CD4^+^PD-1^+^ Cells and Inferior Survival in Patients with Newly Diagnosed Multiple Myeloma. Clin. Cancer Res..

[55] Rana P.S., Soler D.C., Kort J., Driscoll J.J. (2022). Targeting TGF-beta signaling in the multiple myeloma microenvironment: Steering CARs and T cells in the right direction. Front. Cell Dev. Biol..

[56] David C.J., Massagué J. (2018). Contextual determinants of TGFβ action in development, immunity and cancer. Nat. Rev. Mol. Cell Biol..

[57] Choi J., Park J., Cho I., Sheen Y. (2022). Co-treatment with vactosertib, a novel, orally bioavailable activin receptor-like kinase 5 inhibitor, suppresses radiotherapy-induced epithelial-to-mesenchymal transition, cancer cell stemness, and lung metastasis of breast cancer. Radiol. Oncol..

[58] Russ A., Hua A.B., Montfort W.R., Rahman B., Riaz I.B., Khalid M.U., Carew J.S., Nawrocki S.T., Persky D., Anwer F. (2018). Blocking “don’t eat me” signal of CD47-SIRPalpha in hematological malignancies, an in-depth review. Blood Rev..

[59] You T., Hu W., Ge X., Shen J., Qin X. (2011). Application of a novel inhibitor of human CD59 for the enhancement of complement-dependent cytolysis on cancer cells. Cell Mol. Immunol..

[60] van de Donk N., Usmani S.Z. (2018). CD38 Antibodies in Multiple Myeloma: Mechanisms of Action and Modes of Resistance. Front. Immunol..

[61] Wang Y., Zhang Y., Hughes T., Zhang J., Caligiuri M.A., Benson D.M., Yu J. (2018). Fratricide of NK Cells in Daratumumab Therapy for Multiple Myeloma Overcome by Ex Vivo-Expanded Autologous NK Cells. Clin. Cancer Res..

[62] Tang Z., Wang X., Tang M., Wu J., Zhang J., Liu X., Gao F., Fu Y., Tang P., Li C. (2023). Overcoming the On-Target Toxicity in Antibody-Mediated Therapies via an Indirect Active Targeting Strategy. Adv. Sci..

[63] Tyrsin D., Chuvpilo S., Matskevich A., Nemenov D., Römer P.S., Tabares P., Hünig T. (2016). From TGN1412 to TAB08: The return of CD28 superagonist therapy to clinical development for the treatment of rheumatoid arthritis. Clin. Exp. Rheumatol..

[64] Gogesch P., Dudek S., van Zandbergen G., Waibler Z., Anzaghe M. (2021). The Role of Fc Receptors on the Effectiveness of Therapeutic Monoclonal Antibodies. Int. J. Mol. Sci..

[65] Ben Mkaddem S., Benhamou M., Monteiro R.C. (2019). Understanding Fc Receptor Involvement in Inflammatory Diseases: From Mechanisms to New Therapeutic Tools. Front. Immunol..

[66] Alegre M.L., Peterson L.J., Xu D., Sattar H.A., Jeyarajah D.R., Kowalkowski K., Thistlethwaite J.R., Zivin R.A., Jolliffe L., Bluestone J.A. (1994). A non-activating “humanized” anti-CD3 monoclonal antibody retains immunosuppressive properties in vivo. Transplantation.

