# Supplementary material for: Pre-Clinical Assessment of SAR442257, a CD38/CD3xCD28 Trispecific T Cell Engager in Treatment of Relapsed/Refractory Multiple Myeloma
Source: Cells. 2024 May 20;13(10):879. doi: 10.3390/cells13100879 (PMC11120574; doi:10.3390/cells13100879)

Figure S1
Workflow and Titration for Cellular avidity experiments: (a) Sketch of the experimental workflow, (b-d) Titration avidity experiments of U266 and MM1.S. (c+e) Frequency of bound T-cells for different concentrations of SAR442257 for (c) U266 and (e) MM1.S.

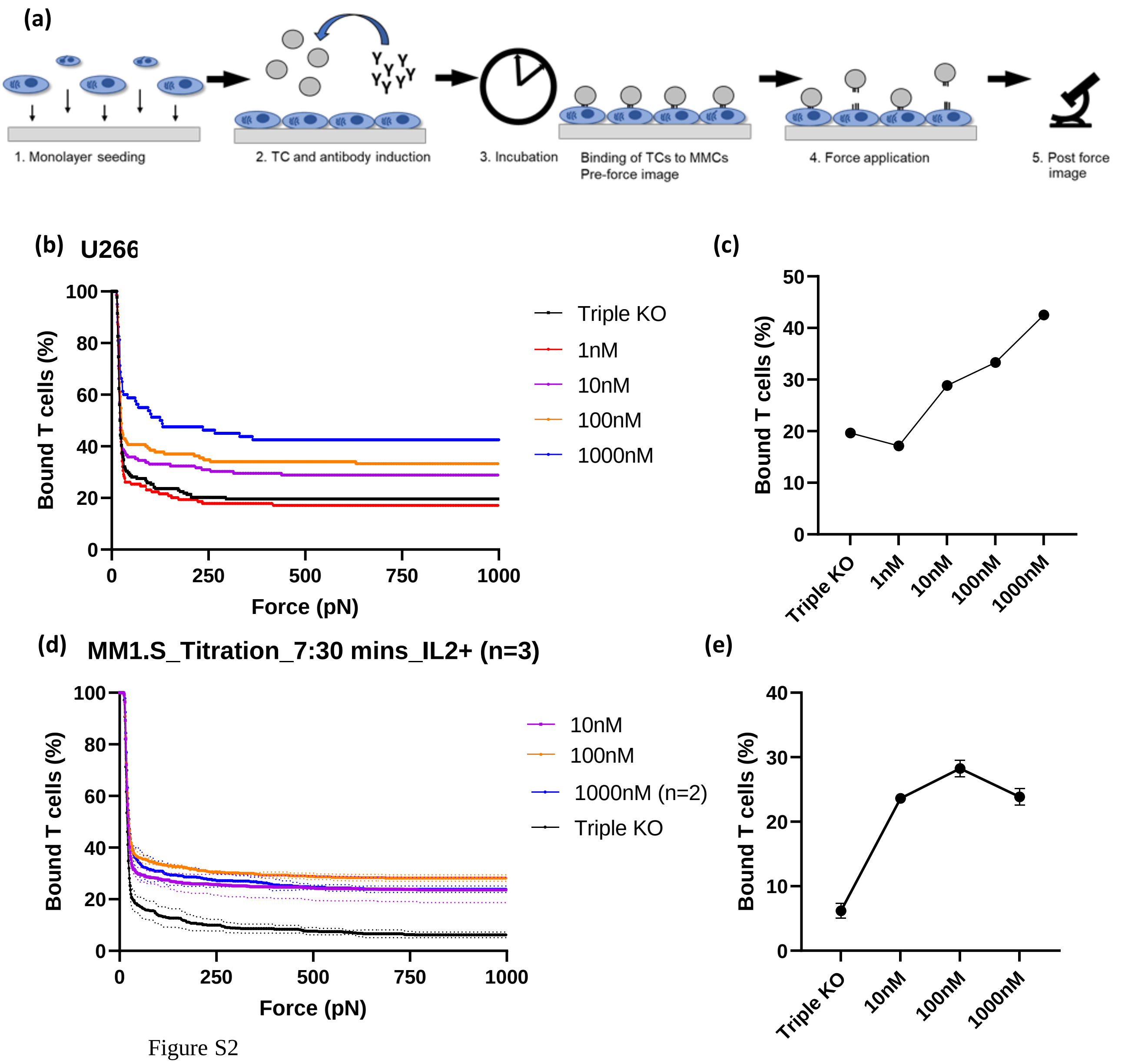

Figure S2
Analysis of SAR442257 potency to induce HMCL death in co-cultures at 1nM.
10 HMCL were characterized for (a) CD38 and (b) CD28 expression

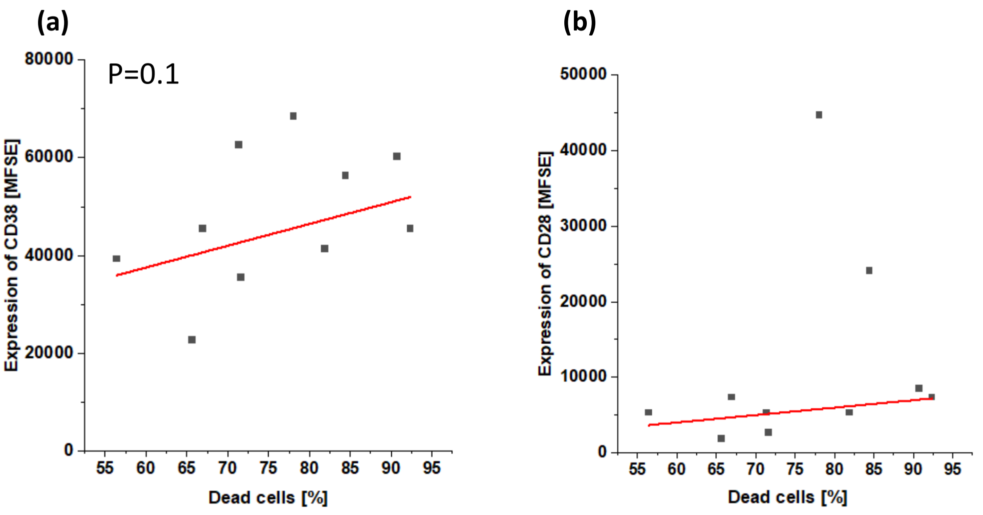

Figure S3

CD38/CD28-target expression of bone marrow microenvironment a) CD3 positive T cells b) early activated T-cells, c) Tregs, d) NK cells. e) CD28 and CD38 target expression on MMCs of 100 NDMM patients and f) 40 RRMM patients refractory to anti-CD38 therapy (black), BiTE (red) or ELO/POM (blue)

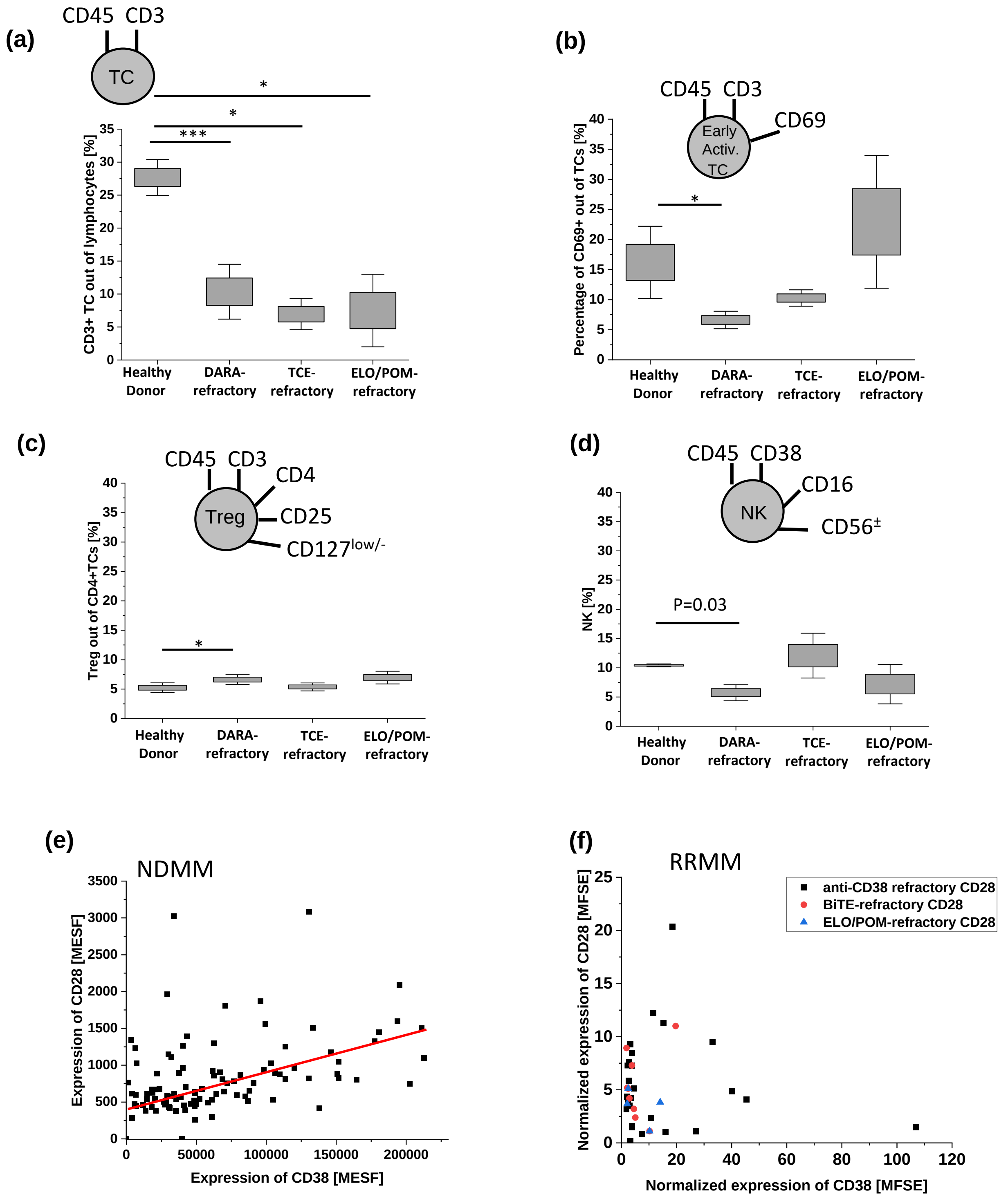

Figure S4
Characterization of CD4 and CD8 TC states, degranulation, and cytokine release: TC subpopulations indicated by respective markers, according to good and poor response groups for (a+c) anti-CD38-refractory patients and (b+d) BiTE-refractory patients

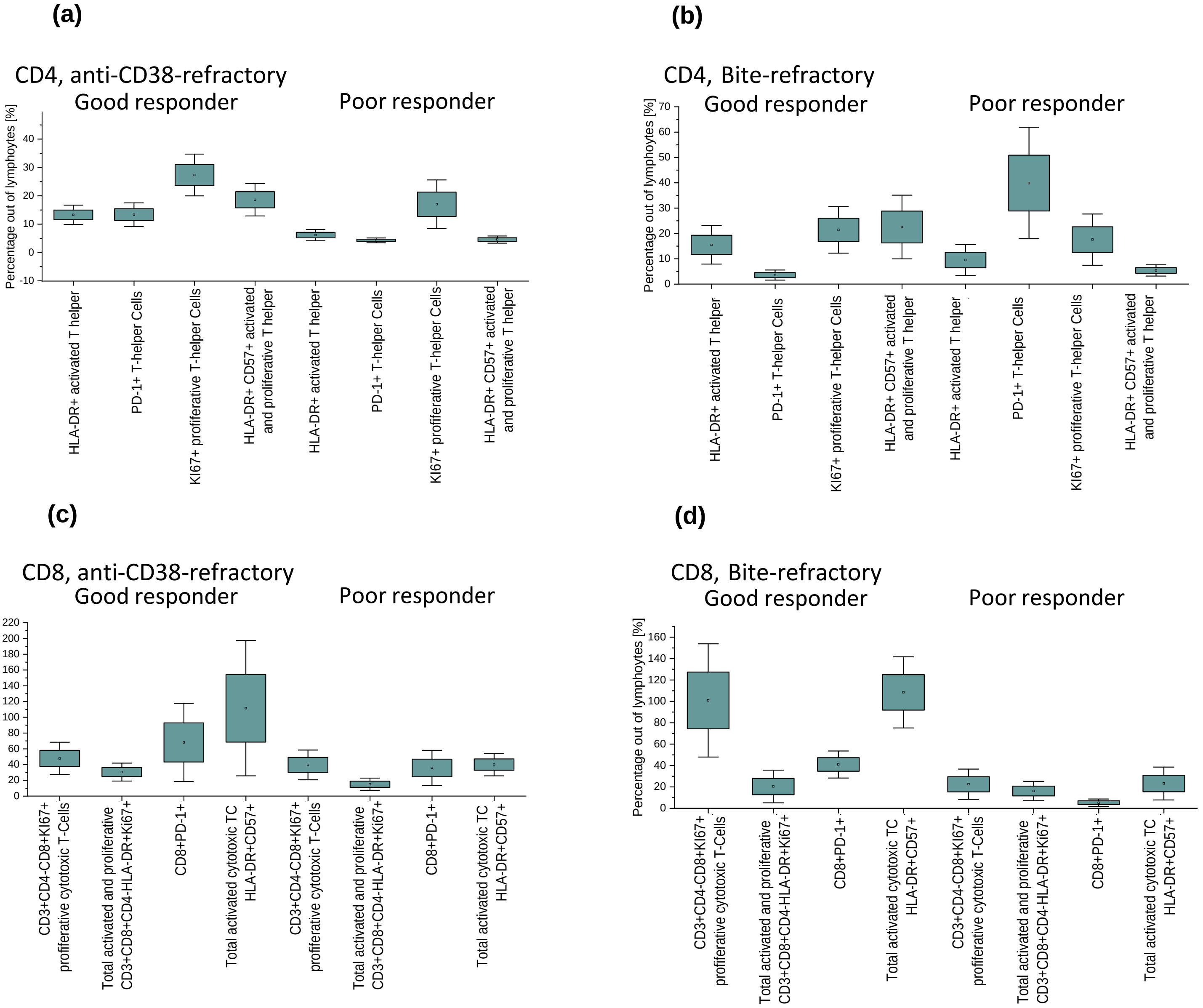

Supplement: Supplementary file 1 [file cells-13-00879-s001.zip › cells-2970486-supplementary.pdf]
